# Propagation-induced revival of entanglement in the angle-OAM bases

**DOI:** 10.1126/sciadv.abn7876

**Published:** 2022-08-05

**Authors:** Abhinandan Bhattacharjee, Mritunjay K. Joshi, Suman Karan, Jonathan Leach, Anand K. Jha

**Affiliations:** ^1^ Department of Physics, Indian Institute of Technology Kanpur, Kanpur UP 208016, India.; ^2^ School of Engineering and Physical Sciences, Heriot-Watt University, Edinburgh EH14 4AS, UK.

## Abstract

Although the continuous-variable position-momentum entanglement of photon pairs produced by parametric down-conversion has applicability in several quantum information applications, it is not suitable for applications involving long-distance propagation. This is because entanglement in the position-momentum bases, as seen through Einstein-Podolsky-Rosen (EPR)–correlation measurements, decays very rapidly with photons propagating away from the source. In contrast, in this article, we show that in the continuous-variable bases of angle–orbital angular momentum (OAM), the entanglement, as seen through EPR-correlation measurements, exhibits a remarkably different behavior. As with the position-momentum bases, initially, the entanglement in the angle-OAM bases also decays with propagation, and after a few centimeters of propagation, there is no angle-OAM entanglement left. However, as the photons continue to travel further away from the source, the entanglement in the angle-OAM bases revives. We theoretically and experimentally demonstrate this behavior and show that angle-OAM entanglement revives even in the presence of strong turbulence.

## Teaser

Entanglement in the angle–orbital angular momentum bases survives at large distances even in the presence of a turbulent medium.

## INTRODUCTION

Quantum entanglement (*1*–*4*) is the key resource behind the advancement of many applications such as quantum imaging (*5*), quantum communication (*6*), quantum information processing (*7*), and quantum computing (*8*). Spontaneous parametric down-conversion (SPDC) is one of the most widely used methods for generating entangled photons in which a pump photon at a higher frequency interacts with a nonlinear crystal and produces two separate photons at lower frequencies called the signal and idler photons. The entanglement of down-converted photons has been extensively studied in the discrete finite-dimensional bases such as polarization (*9*), time-bin (*10*, *11*), and orbital angular momentum (OAM) (*12*, *13*) as well as in the continuous-variable bases such as position-momentum (*14*–*16*), angle-OAM (*17*), radial position–radial momentum (*18*), and time-energy (*19*, *20*). Although there are several ways of quantifying two-photon entanglement in two-dimensional bases (*21*), there is no quantifier for more than two-dimensional bases and continuous-variable bases in which cases one can talk only in terms of entanglement certifiers (*4*). For continuous-variable bases, there are several entanglement certifiers such as the Einstein-Podolsky-Rosen (EPR) criterion (*1*, *14*–*17*, *22*), partial transpose (*23*, *24*), and Rényi entropy (*25*, *26*). Among these certifiers, the EPR criterion is the most widely used one and is used even beyond photonic quantum systems (*27*, *28*).

The practical implementation of quantum information tasks requires entanglement to be sustained over long distances and in turbulent environments. The feasibility of using entanglement in the finite-dimensional bases for long-distance quantum-information applications has been demonstrated in several experimental works (*29*–*32*). However, the suitability of entanglement in the continuous-variable bases for long-distance applications has not been established so far. Among the continuous-variable bases, position-momentum bases have been extensively investigated for its applicability in several applications such as quantum imaging (*33*–*36*), quantum holography (*37*, *38*), quantum metrology (*39*), and quantum secure communication (*40*, *41*). Although position-momentum entanglement has found uses in many of these applications, it has not been found suitable for applications involving long-distance propagation. This is because of the fact that as the photons propagate away from the down-conversion crystal, the entanglement in the position-momentum bases, seen through EPR correlation measurements, decays very rapidly (*42*, *43*), and this effect becomes worse in the presence of turbulent environments.

In this article, we explore entanglement of down-converted photons in the continuous-variable bases of angle and OAM. We certify entanglement through EPR correlation measurements (*14*–*17*, *22*, *44*) and demonstrate that the entanglement of down-converted photons in the angle-OAM bases exhibits a different behavior than the entanglement in the position-momentum bases. Just as in the case of position-momentum bases, initially, the angle-OAM entanglement decays with propagation, but as the photons continue to travel further away from the source, the entanglement in the angle-OAM bases comes back. We refer to this behavior as the propagation-induced revival of entanglement in the angle-OAM bases. We theoretically and experimentally demonstrate this behavior and show that this propagation-induced revival takes place even in the presence of strong turbulence.

## RESULTS

We present a quantitative analysis of the propagation of conditional position and angle uncertainties of the signal photon in SPDC. For a Gaussian pump with beam waist at the crystal plane *z* = 0, the two-photon wave function in the position basis at a propagation distance *z* is given by (*15*, *42*, *43*) $\psi (\rho_{s},\rho_{i},z)=A\text{exp}\;[-\frac{{|\rho_{s}+\rho_{i}|}^{2}}{4w{\left(z\right)}^{2}}]\text{exp}\;[-\frac{{|\rho_{s}-\rho_{i}|}^{2}}{4\sigma {\left(z\right)}^{2}}]e^{i\phi (\rho_{s},\rho_{i},z)}$, where ρ_s_ ≡ (*x*_s_, *y*_s_) and ρ_i_ ≡ (*x*_i_, *y*_i_) are the transverse positions of the signal and idler photons, respectively at *z*, and where $w(z)=w_{0}\sqrt{1+z^{2}/(k^{2}w_{0}^{4})}$, $\sigma (z)=\sigma_{0}\sqrt{1+z^{2}/(k^{2}\sigma_{0}^{4})}$, and *k* = π/λ_p_. In addition, *w*_0_ is the pump beam waist at *z* = 0, $\sigma_{0}=\sqrt{0.455L\lambda_{p}/2\pi }$, *L* is the length of the crystal, λ_p_ is the wavelength of the pump field, and $e^{i\phi (\rho_{s},\rho_{i},z)}$ is a phase factor. The two-photon position probability distribution function *P*(ρ_**s**_, ρ_i_; *z*) = ∣ψ(ρ_s_, ρ_i_; *z*)^2^∣ at *z* is therefore given by

$$P(\rho_{s},\rho_{i},z)={|A|}^{2}\text{exp}\;[-\frac{{|\rho_{s}+\rho_{i}|}^{2}}{2w{\left(z\right)}^{2}}]\text{exp}\;[-\frac{{|\rho_{s}-\rho_{i}|}^{2}}{2\sigma {\left(z\right)}^{2}}]$$ (1)

**Fig. 1.**
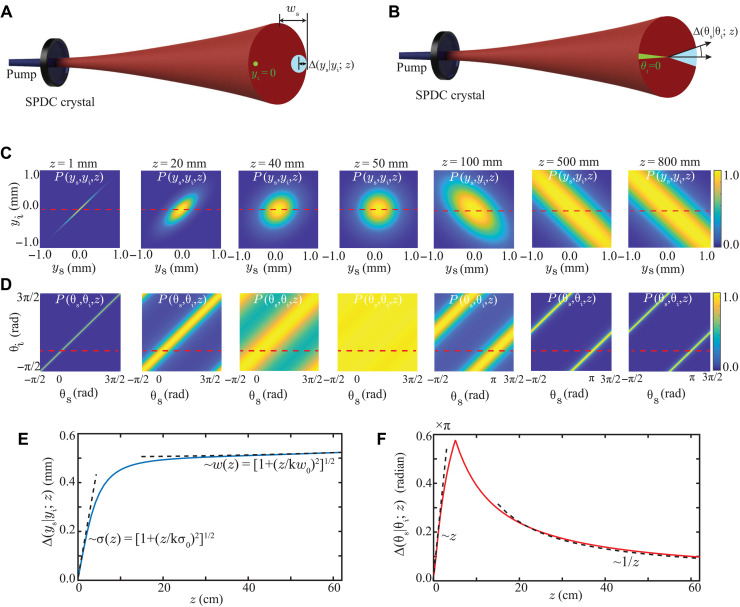
Propagation of conditional position and angle uncertainties. (**A** and **B**) Schematic illustrations of conditional position and conditional angle uncertainties. (**C** and **D**) Numerically evaluated two-photon position probability distribution function *P*(*y*_s_, *y*_i_; *z*) and the two-photon angle probability distribution function *P*(θ_s_, θ_i_; *z*), respectively, at various *z* values. (**E**) Numerically calculated conditional position uncertainty Δ(*y*_s_∣*y*_i_; *z*) as a function of *z*. The two dashed lines show the *z*-scaling of the uncertainty in the near- and far-field regions. (**F**) Numerically calculated conditional angle uncertainty Δ(θ_s_∣θ_i_; *z*) as a function of *z*. The two dotted lines show the *z*-scaling of the uncertainty in the near- and far-field regions.

For a fixed idler position, say in the *y* direction, the two-photon position probability distribution is referred to as the conditional position probability distribution of the signal photon at *z* and is denoted by *P*(*y*_s_∣*y*_i_; *z*). The standard deviation of *P*(*y*_s_∣*y*_i_; *z*) is referred to as the conditional position uncertainty Δ(*y*_s_∣*y*_i_; *z*) of the signal photon (see Fig. 1A for illustrations). Similarly, by writing the two-photon wave function ψ(ρ_s_, ρ_i_; *z*) in the transverse momentum basis, one can calculate conditional momentum uncertainty Δ(*p*_*y*s_∣*p*_*y*i_; *z*) of the signal photon at *z*. According to the EPR criterion of entanglement, if the product Δ(*y*_s_∣*y*_i_; *z*)Δ(*p*_*y*s_∣*p*_*y*i_; *z*) < 0.5 ħ, then the two photons are entangled in the position-momentum bases (*1*, *14*).

In addition to the position-momentum bases, the down-converted photons are rendered entangled in the angle-OAM bases as well. Using Eq. 1 and the transformations ρ_s_ = (*r*_s_ cos θ_s_, *r*_s_ sin θ_s_) and ρ_i_ = (*r*_i_ cos θ_i_, *r*_i_ sin θ_i_), we can write the two-photon angle probability distribution function *P*(θ_s_, θ_i_; *z*) as

$$P(\theta_{s},\theta_{i};z)=\iint r_{s}r_{i}P(r_{s},\theta_{s},r_{i},\theta_{i};z)dr_{s}dr_{i}$$ (2)

where (*r*_s_, θ_s_) and (*r*_i_, θ_i_) are the polar coordinates of the signal and idler photons. Using *P*(θ_s_, θ_i_; *z*), one can obtain the conditional angle probability distribution function *P*(θ_s_∣θ_i_; *z*) and thereby the conditional angle uncertainty Δ(θ_s_∣θ_i_; *z*) of the signal photon (see Fig. 1B for illustrations). Denoting the conditional OAM uncertainty by Δ(*l_s_*∣*l*_i_; *z*), we write the EPR criterion for entanglement in the angle-OAM bases as Δ(θ_s_∣θ_i_; *z*)Δ(*l*_s_∣*l*_i_; *z*) < 0.5 ħ[1 − 2π*P*(θ_0_∣θ_i_; *z*)] (*17*, *45*), where *P*(θ_0_∣θ_i_; *z*) represents the conditional probability of detecting the signal photon at the boundary θ_s_ = θ_0_.

Now, using Eqs. 1 and 2 and the relevant experimental parameters *w*_0_ = 507 μm, *L* = 5 mm, and λ_p_ = 355 nm, we numerically evaluate *P*(*y*_s_, *y*_i_; *z*) and *P*(θ_s_, θ_i_; *z*) at different propagation distances *z* and plot them in Fig. 1 (C and D, respectively). In plotting *P*(*y*_s_, *y*_i_; *z*) and *P*(θ_s_, θ_i_; *z*), we scale them to make their maximum values equal to one. Next, by fixing *y*_i_ = 0 in *P*(*y*_s_, *y*_i_; *z*) and θ_i_ = 0 in *P*(θ_s_, θ_i_; *z*), we calculate *P*(*y*_s_∣*y*_i_; *z*) and *P*(θ_s_∣θ_i_; *z*) and thereby the conditional position uncertainty Δ(*y*_s_∣*y*_i_; *z*) and the conditional angle uncertainty Δ(θ_s_∣θ_i_; *z*), and we plot them in Fig. 1 (E and F, respectively). From the plots in Fig. 1 (E and F), we find that as the down-converted photons propagate away from the crystal, the conditional position uncertainty increases monotonically. However, the conditional angle uncertainty increases initially but later begins to decrease monotonically. (See sections S1 and S2 for more detailed analysis and numerical simulations.) We emphasize that to observe revival of entanglement in the angle-OAM bases, one needs to collect the entire transverse field. If a part of the field is rejected because of the finite size of the detector, then that will result in a loss of entanglement. We have carried our numerical simulation of the effects of a finite-size detector on the propagation of angle-OAM entanglement (see section S9 for more details.) We have found that increasing the detector size increases the propagation distance up to which the angle-OAM entanglement remains intact. Therefore, one can decide the minimum aperture size for a given application based on the distance up to which one needs the entanglement in the angle-OAM bases to be intact.

Although it is very difficult to derive the general analytical expressions for the conditional position and angle uncertainties as a function of *z*, we derive expressions for how the conditional uncertainties scale with *z* in the near- and far-field regions. The two dotted lines in Fig. 1E show how the conditional position uncertainty Δ(*y*_s_∣*y*_i_; *z*) scales with *z* in the near and far fields. We find that Δ(*y*_s_∣*y_i_*; *z*) increases as σ(*z*) in the near field, while it increases as *w*(*z*) in the far field. The two dotted lines in Fig. 1F show how the conditional angle uncertainty Δ(θ_s_∣θ_i_; *z*) scales with *z*. We find that while Δ(θ_s_∣θ_i_; *z*) increases as *z* in the near-field regions, it decreases as 1/*z* in the far-field regions. (For the detailed theoretical calculations of the scaling laws, see sections S1.2 and S1.3).

The plots in Fig. 1 (E and F) depict how the conditional position uncertainty Δ(*y*_s_∣*y*_i_; *z*) and conditional angle uncertainty Δ(θ_s_∣θ_i_; *z*) change as a function of *z*. We note that the conditional momentum and OAM uncertainties Δ(*p*_*y*s_∣*p*_*y*i_; *z*) and Δ(*l*_s_∣*l*_i_; *z*) remain constant as a function of *z* because of the conservations of momentum and OAM, respectively, in SPDC. As a result, the functional dependence of Δ(*y*_s_∣*y*_i_; *z*)Δ(*p*_*y*s_∣*y*_*y*i_; *z*) and Δ(θ_s_∣θ_i_; *z*)Δ(*l*_s_∣*l*_i_; *z*) on *z* is same as that of Δ(*y*_s_∣*y*_i_; *z*) and Δ(θ_s_∣θ_i_; *z*), respectively. Therefore, the entanglement in the position-momentum bases as seen through EPR correlation product was found to decay very rapidly as a function of *z*, and after a few centimeters of propagation, there is no entanglement left in the position-momentum bases. On the other hand, although there is no entanglement left in the angle-OAM bases after a few centimeters of propagation just as in the case of position-momentum bases, the entanglement in the angle-OAM bases comes back after further propagation. We refer to this as the propagation-induced revival of entanglement in the angle-OAM bases. Although in our analysis, so far, we have used EPR uncertainty product as an entanglement witness, we observe qualitatively the same features in the propagation of angle-OAM entanglement with another witness, entanglement of formation (*46*, *47*) (see section S10 for details).

We note that the propagation of entangled photons can be modeled in terms of local unitary transformations, and therefore, it cannot change the total entanglement of the two-photon state (*48*). The simulations we conducted do not depict how the total entanglement of the two-photon state changes upon propagation. They instead depict how the total entanglement manifests in the position-momentum and angle-OAM bases upon propagation. Such propagation-dependent manifestations of entanglement in different bases have also been reported in the amplitude and phase bases (*49*, *50*). In the context of the amplitude and phase bases, it has been shown, under the very restrictive double-Gaussian approximation for the two-photon wave function, that although the entanglement in amplitude and phase changes individually as a function of *z*, the sum of the entanglements in the amplitude and phase remains constant (*51*). However, it has so far not been possible to show this for a two-photon state without the double-Gaussian approximation or for a mixed two-photon state. In our present work, we find that in the far field, entanglement revives in the angle-OAM bases but not in the position-momentum bases. This fact could be used for long-distance applications involving angle-OAM entanglement. Nonetheless, how the total entanglement of a general two-photon state could be expressed as an invariant of propagation in terms of its manifestations in the position-momentum, angle-OAM, and other bases still remains an open question.

**Fig. 2.**
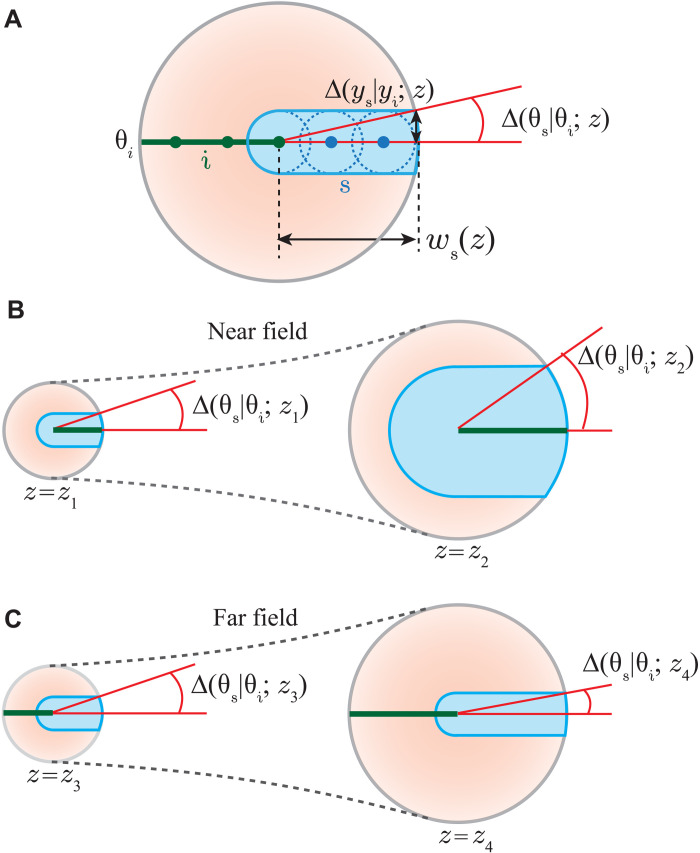
Pictorial illustration of propagation of conditional angle uncertainty. (**A**) The cross section of the down-converted field as it propagates. For a line of idler positions, shown by green line, the signal photon has the probability of being found in the blue shaded region. The conditional angle uncertainty Δ(θ_**s**_∣θ_**i**_; *z*) is of the order of Δ(*y*_**s**_∣*y*_**i**_; *z*)/*w*_**s**_(*z*), where Δ(*y*_**s**_∣*y*_**i**_; *z*) is the conditional position uncertainty of the signal photon and *w*_**s**_(*z*) is the size of the down-converted field. (**B**) and (**C**) illustrate that the conditional angle uncertainty increases as a function of *z* in the near field, while in the far field, it decreases as a function of *z*.

Next, we present a pictorial illustration of how the conditional angle uncertainty decreases upon propagation in the far field causing entanglement in the angle-OAM bases to revive. Figure 2A shows the cross section of the down-converted field as it propagates. For an idler position *y*_i_, shown by a green dot, the signal has the appreciable probability to be found in a circular area of radius Δ(*y*_s_∣*y*_i_; *z*), which is the conditional position uncertainty of the signal photon at *z*. Therefore, if we have a line of idler positions, shown by green line, the signal photon has the appreciable probability of being found in the blue shaded region. This green line represents the angular position θ_i_ = 0 of the idler. The corresponding conditional angle uncertainty Δ(θ_s_∣θ_i_; *z*) of the signal photon can be estimated by averaging the angle over the radial extent of the down-converted field from the center up to beam width *w*_s_(*z*) of the field. However, to a very good approximation, we can take the conditional angle uncertainty Δ(θ_s_∣θ_i_; *z*) to be of the order of Δ(*y*_s_∣*y*_i_; *z*)/*w*_s_. Figure 1E shows how Δ(*y*_s_∣*y*_i_; *z*) changes as a function of *z*, and fig. S3 in the Supplementary Materials shows how *w*_s_(*z*) changes as a function of *z*. We find that in the near field, Δ(*y*_s_∣*y*_i_; *z*) increases much faster compared to *w*_s_(*z*), while in the far field, it increases much slower compared to *w*_s_(*z*). Because of this, as a function of *z*, the conditional angle uncertainty Δ(θ_s_∣θ_i_; *z*) increases in the near field, while it decreases in the far field (illustrated in Fig. 2, B and C).

**Fig. 3.**
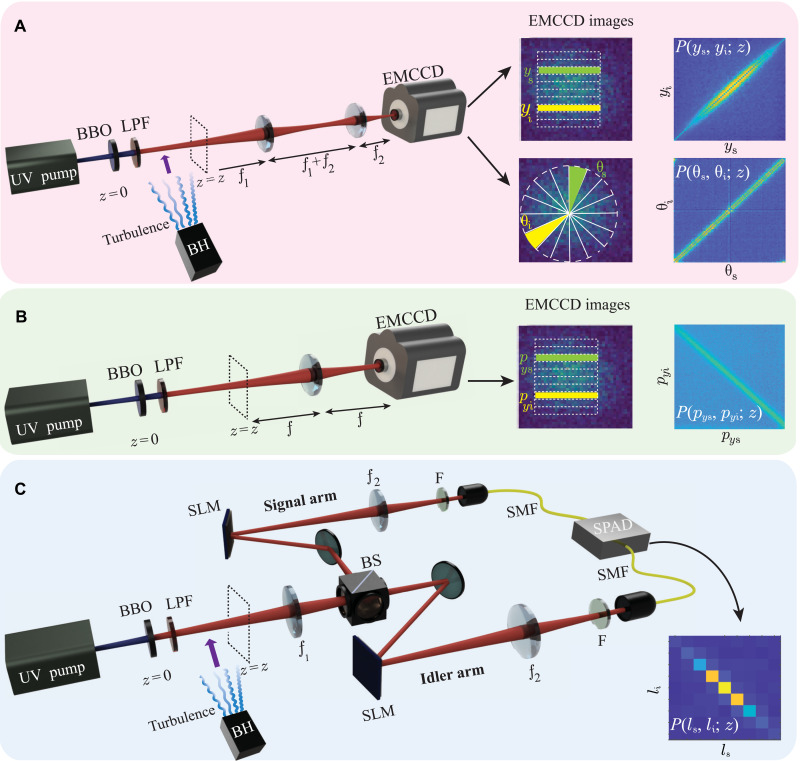
Experimental setup. (**A**) Schematic of the experimental setup for measuring position and angle coincidences. Inset shows the EMCCD images of the SPDC field and the corresponding two-photon position and angle probability distribution functions. (**B**) Schematic of the experimental setup for measuring the two-photon momentum probability distribution function. Inset shows EMCCD images of the SPDC field and the corresponding two-photon momentum probability distribution function. (**C**) Schematic of the experimental setup for measuring OAM coincidence and the OAM correlation. LPF, long-pass filter; BS, beam splitter; SLM, spatial light modulator; SMF, single-mode fiber; F, interference filter. The BH is used for generating turbulence, and it is switched on in the path of the SPDC field when studying the effect of turbulence on entanglement propagation.

Figure 3 shows the schematic of the experimental setup for measuring the two-photon probability distribution functions *P*(*y*_s_, *y*_i_; *z*), *P*(*p*_*y*s_, *p*_*y*i_; *z*), *P*(θ_s_, θ_i_; *z*), and *P*(*l*_s_, *l*_i_; *z*) through coincidence measurements of the two photons. An ultraviolet (UV) continuous wave (CW) Gaussian pump (Coherent Genesis STM UV laser) of wavelength λ_p_ = 355 nm, beam waist *w*_0_ = 507 μm is incident on a 5 mm–by–5 mm–by–5 mm β-barium borate (BBO) crystal. The crystal is cut in a manner that it produces signal and idler photons with collinear type I phase-matching condition. A long-pass filter is placed after the crystal to block the UV pump. We use an Andor iXon Ultra-897 electron-multiplied charge-coupled device (EMCCD) camera that has a 512 × 512 pixel grid with each pixel being 16 μm by 16 μm in size. A 10-nm bandpass filter centered at 710 nm is used to detect the down-converted photons. The blower heater (BH) produces turbulence by blowing hot air, and it is switched on during our experiments involving turbulence.

**Fig. 4.**
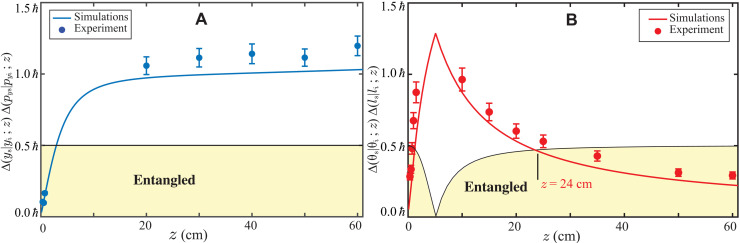
Propagation of entanglement in the position-momentum and angle-OAM bases. (**A**) Conditional position-momentum uncertainty product Δ(*y*_s_∣*y*_i_; *z*)Δ(*p*_s*y*_∣*p*_i*y*_; *z*) as a function of the propagation distance *z*. The solid dots are the experimental results, and the solid line is the numerical simulation. (**B**) Conditional angle-OAM uncertainty product Δ(θ_s_∣θ_i_; *z*)Δ(*l*_s_∣*l*_i_; *z*) as a function of the propagation distance *z*. The solid dots are the experimental results, and the solid line is the numerical simulation. As indicated on the plot, the theoretical prediction for entanglement revival is at *z* = 24 cm, while we observe it experimentally at about *z* = 28 cm.

For the coincidence measurements of *P*(*y*_s_, *y*_i_; *z*), *P*(*p*_*y*s_, *p*_*y*i_; *z*), and *P*(θ_s_, θ_i_; *z*), we use an EMCCD camera (*52*, *53*), as depicted in Fig. 3 (A and B). For measuring *P*(*y*_s_, *y*_i_; *z*) and *P*(θ_s_, θ_i_; *z*) and thereby the corresponding uncertainties Δ(*y*_s_∣*y*_i_; *z*) and Δ(θ_s_∣θ_i_; *z*), we image the transverse plane at *z* onto the EMCCD camera plane using a *4f*-imaging system, as depicted in Fig. 3A. For measuring *P*(*p*_*y*s_, *p*_*y*i_; *z*) at *z*, we use a *2f* imaging system and keep the EMCCD camera plane at the Fourier plane of the transverse plane at *z*, as depicted in Fig. 3B. We then measure the two-photon position probability distribution function at the EMCCD camera plane, which is proportional to the two-photon momentum probability distribution function *P*(*p*_*y*s_, *p*_*y*i_; *z*) at *z*. The conditional momentum uncertainty Δ(*p*_*y*s_∣*p*_*y*i_; *z*) is obtained by multiplying the conditional position uncertainty at the EMCCD plane by *kħ*/*f*, where *f* is the focal length of the lens. (For details regarding measurement techniques and results, see sections S3 to S5). For the coincidence measurements of the two-photon OAM probability distribution *P*(*l*_s_, *l*_i_; *z*), we make use of two electronically gated single-photon avalanche diode (SPAD) detectors (*17*), as depicted in Fig. 3C. We image the transverse plane at *z* onto the spatial light modulators (SLMs) kept in the signal and idler arms. Specific holograms are displayed onto both the SLMs, and then, the signal and idler SLM planes are imaged onto the input facets of single-mode fibers (SMFs) kept in the signal and idler arms. The combination of the hologram and SMF in each arm projects the input field into a particular OAM mode, which then gets detected by the SPAD detector through the SMF. An electronic coincidence circuit then yields the coincidence counts. By displaying different holograms on the SLMs, we measure the two-photon OAM probability distribution. Next, we report our measurements of the condition uncertainty products Δ(*y*_s_∣*y*_i_; *z*)Δ(*p*_*y*s_∣*p*_*y*i_; *z*) and Δ(θ_s_∣θ_i_; *z*)Δ(*l*_s_∣*l*_i_; *z*) at various *z* values in the absence of turbulence. In our experiments, we measure Δ(*p*_*y*s_∣*p*_*y*i_; *z*) to be 2.13ħ ± 0.1ħ mm^−1^, which is in good agreement with the value 1.97 ħ mm^−1^ calculated using Eq. 1. We note that with our theoretical modeling based on the Gaussian pump beam, Δ(*l*_s_∣*l*_i_; *z*) should be zero. However, because of the imperfection in the spatial profile of the beam and other background issues, Δ(*l*_s_∣*l*_i_; *z*) is always finite in realistic experimental situations. We measure Δ(*l*_s_∣*l*_i_; *z*) to be 0.72 ± 0.04 ħ radian^−1^ and use this in our experiments (see section S2 of the Supplementary Materials for more details). Last, we measure Δ(*y*_s_∣*y*_i_; *z*) and Δ(θ_s_∣θ_i_; *z*) at various *z* values and plot the conditional uncertainty products Δ(*y*_s_∣*y*_i_; *z*)Δ(*p*_*y*s_∣*p*_*y*i_; *z*) and Δ(θ_s_∣θ_i_; *z*)Δ(*l*_s_∣*l*_i_; *z*) as a function of *z* in Fig. 4 (A and B, respectively). Figure 4 (A and B) also shows the numerical simulations using Eqs. 1 and 2. We find that there is no entanglement left in either the position-momentum or the angle-OAM bases at few centimeters away from the down-conversion crystal. However, while the entanglement in the position-momentum bases never revives, the entanglement in the angle-OAM bases, as simulated numerically, revives after the photons have propagated 24 cm away from the crystal; experimentally, we find this distance to be about 28 cm. After the revival, the entanglement in the angle-OAM bases stays intact.

**Fig. 5.**
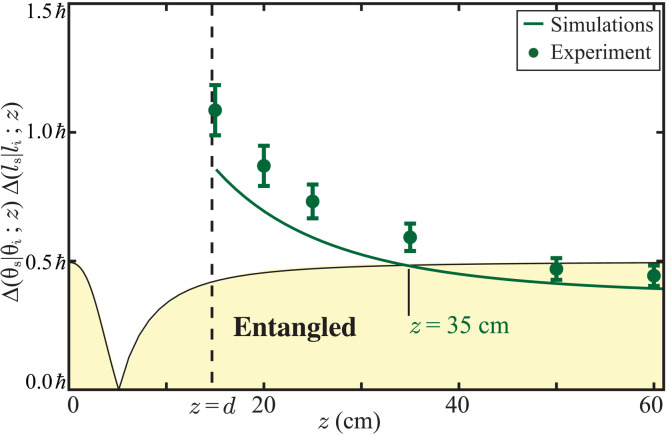
Entanglement revival in the presence of turbulence. Conditional angle-OAM uncertainty product Δ(θ_s_∣θ_i_; *z*)Δ(*l*_s_∣*l*_i_; *z*) as a function of the propagation distance *z* in the presence of a turbulent medium. As indicated on the plot, in the presence of turbulence, the theoretical prediction for entanglement revival is at *z* = 35 cm, while we observe it experimentally at about 45 cm.

We next investigate whether the propagation-induced entanglement revival takes place in turbulent environments, which is quite often the limiting factor in the practical implementations of many entanglement-based applications. For this, we repeat our experiments depicted in Fig. 3 (A and B) in the angle-OAM bases with the BH switched on and kept at *z* = 15 cm to introduce turbulence (*54*) in the path of the down-converted photons. We experimentally measure the product Δ(θ_s_∣θ_i_; *z*)Δ(*l*_s_∣*l*_i_; *z*) at different propagation distances ranging from *z* = 15 cm to *z* = 60 cm and plot them in Fig. 5. The solid line represents the numerically calculated value of the uncertainty product. For the detailed modeling of turbulence (*55*) and the numerical calculations of Δ(θ_s_∣θ_i_; *z*)Δ(*l*_s_∣*l*_i_; *z*) in the presence of turbulence, see sections S6 and S7 of the Supplementary Materials. Our numerical simulations show that in the presence of turbulence, the angle-OAM entanglement revives at *z* = 35 cm; experimentally, we find this distance to be about 45 cm. Therefore, we find that although turbulence does affect entanglement in the angle-OAM bases in an adverse manner, its effect can be completely undone by just propagating the photons further away by some distance.

We find a very good qualitative agreement between our experiment results and numerical simulations reported inFigs. 4 and 5. However, quantitatively, there is a systematic difference between the experimental results and simulations. We attribute this mostly to the unavoidable background noise floor involved in the EMCCD-based coincidence measurements. The noise floor results in the overestimation of conditional uncertainties causing all the experimental data points to have a systematic difference with the simulations. In addition, in our analysis, we approximate the two-photon wave function in Eq. 1 to have a double-Gaussian form. However, it is known that this is not a very good approximation (*51*). Nevertheless, we make this approximation otherwise the numerical simulations become extremely time consuming. This double-Gaussian approximation also adds to the systematic difference between the simulations and the experimental results. We further note that it is relatively easier to measure Δ(*y*_s_∣*y*_i_; *z*)Δ(*p*_*y*s_∣*y*_*y*i_; *z*) and Δ(θ_s_∣θ_i_; *z*)Δ(*l*_s_∣*l*_i_; *z*) close to the crystal (*z* < 3 cm) or in the far field (*z* > 10 cm). However, because of the signal-to-noise limitations of the EMCCD camera, it is not possible to make measurements in the intermediate regions. (For details on these measurements, see sections S4 and S5 of the Supplementary Materials.)

## DISCUSSION

In conclusion, using the two-photon field produced by SPDC, we have reported experimental observations of propagation-induced revival of entanglement in the angle-OAM bases. We have demonstrated this entanglement revival even in the presence of turbulence, the only effect of which is to increase the propagation distance for revival. Once revived, the two photons remain entangled up to an arbitrary propagation distance in the angle-OAM bases. We note that the entanglement revival strategies in turbulence or random media are usually based on adaptive optics techniques (*31*, *56*, *57*), which involve feedback mechanisms and thus are quite difficult to implement. On the other hand, in our work, we have shown that entanglement in the angle-OAM bases can be revived simply by further propagating the two-photon field by some distance, without having to use any adaptive optics techniques. Thus, the angle-OAM bases brings in an independent parameter—the propagation distance—for entanglement revival in turbulent environments and can therefore have important implications for long-distance quantum information applications. In this context, we note that most of the quantum imaging and metrology applications that are based on using entanglement in the position-momentum bases do so by imaging the *z* = 0 plane of the down-conversion crystal onto a suitable *z* plane in the far field (*33*–*39*). However, this severely limits the ways in which entanglement in the position-momentum bases could be used. On the other hand, because the entanglement in the angle-OAM bases is naturally present in the far field, it does not suffer from such limitations and could therefore prove very useful for quantum imaging and metrology applications. We also note that although we have demonstrated entanglement revival over laboratory scale only, the effect could very well be demonstrated over a kilometer scale, because it has been experimentally demonstrated that even a 3-km atmospheric turbulence has only a small effect on OAM mode superpositions (*58*). This implies that even a few-kilometers-thick atmospheric turbulence should not have too bad an effect on two-photon OAM mode superpositions and thus on the revival effect.
