# Supplementary material for: Propagation-induced revival of entanglement in the angle-OAM bases
Source: Sci Adv. 2022 Aug 5;8(31):eabn7876. doi: 10.1126/sciadv.abn7876 (PMC9355354; doi:10.1126/sciadv.abn7876)
Supplement: Supplementary file 1 — Supplementary Text Figs. S1 to S11 [file sciadv.abn7876_sm.pdf]

Science Advances
AAAS

Supplementary Materials for

Propagation-induced revival of entanglement in the angle-OAM bases

Abhinandan Bhattacharjee *et al.*

Corresponding author: Abhinandan Bhattacharjee, arko.hkd@gmail.com; Anand K. Jha, akjha@iitk.ac.in

Sci. Adv. **8**, eabn7876 (2022)
DOI: 10.1126/sciadv.abn7876

This PDF file includes:

Supplementary Text
Figs. S1 to S11

1 Calculation of conditional position and angle uncertainties

1.1 Derivation of the formulas

For a Gaussian pump with beam waist at the crystal plane $z = 0$, the two-photon wavefunction in the position basis at the crystal plane $z = 0$ is given by (*15, 43, 44*):

$$\psi(\boldsymbol{\rho}_s, \boldsymbol{\rho}_i; 0) = A \exp\left[-\frac{|\boldsymbol{\rho}_s + \boldsymbol{\rho}_i|^2}{4w_0^2}\right] \exp\left[-\frac{|\boldsymbol{\rho}_s - \boldsymbol{\rho}_i|^2}{4\sigma_0^2}\right], \tag{1}$$

where $\boldsymbol{\rho}_s \equiv (x_s, y_s)$ and $\boldsymbol{\rho}_i \equiv (x_i, y_i)$ are the transverse positions of the signal and idler photons, respectively at z, $k = \pi/\lambda_p$, $|\boldsymbol{\rho}_s| = \rho_s$, and $|\boldsymbol{\rho}_i| = \rho_i$. Also, w_0 is the pump beam waist at $z = 0$, $\sigma_0 = \sqrt{0.455L\lambda_p/2\pi}$, L is the length of the crystal, and λ_p is the wavelength of the pump field. Using the two-photon wave-function $\psi(\boldsymbol{\rho}_s, \boldsymbol{\rho}_i; 0)$ at $z = 0$, we calculate the two-photon wave-function $\psi(\boldsymbol{\rho}_s, \boldsymbol{\rho}_i; z)$ at z and thereby the two-photon position probability distribution function $P(\boldsymbol{\rho}_s, \boldsymbol{\rho}_i; z) = |\psi^*(\boldsymbol{\rho}_s, \boldsymbol{\rho}_i; z)\psi(\boldsymbol{\rho}_s, \boldsymbol{\rho}_i; z)|$ at z, which can be shown to be

$$P(\boldsymbol{\rho}_s, \boldsymbol{\rho}_i; z) = |A|^2 \exp\left[-\frac{|\boldsymbol{\rho}_s + \boldsymbol{\rho}_i|^2}{2w(z)^2}\right] \exp\left[-\frac{|\boldsymbol{\rho}_s - \boldsymbol{\rho}_i|^2}{2\sigma(z)^2}\right]. \tag{2}$$

where $w(z) = w_0\sqrt{1 + z^2/(k^2 w_0^4)}$ and $\sigma(z) = \sigma_0\sqrt{1 + z^2/(k^2\sigma_0^4)}$.

In addition to the position-momentum bases the down-converted photons are rendered entangled in the angle-OAM bases as well. We obtain the two-photon angle probability distribution by writing $P(\boldsymbol{\rho}_s, \boldsymbol{\rho}_i; z)$ of Eq. (2) in the polar coordinates using the transformations $\boldsymbol{\rho}_s = (r_s\cos\theta_s, r_s\sin\theta_s)$ and $\boldsymbol{\rho}_i = (r_i\cos\theta_i, r_i\sin\theta_i)$, where (r_s, θ_s) and (r_i, θ_i) are the polar coordinates of the signal and idler photons at z, etc. We therefore get:

$$\begin{aligned}
&|\boldsymbol{\rho}_s + \boldsymbol{\rho}_i|^2 = r_s^2 + r_i^2 + 2r_s r_i\cos(\theta_s - \theta_i), \\
&|\boldsymbol{\rho}_s - \boldsymbol{\rho}_i|^2 = r_s^2 + r_i^2 - 2r_s r_i\cos(\theta_s - \theta_i), \\
&P(r_s, \theta_s, r_i, \theta_i; z) = |A|^2 \exp\left[-\frac{r_s^2 + r_i^2 + 2r_s r_i\cos(\theta_s - \theta_i)}{2w(z)^2}\right] \\
&\qquad\qquad \times \exp\left[-\frac{r_s^2 + r_i^2 - 2r_s r_i\cos(\theta_s - \theta_i)}{2\sigma(z)^2}\right].
\end{aligned} \tag{3}$$

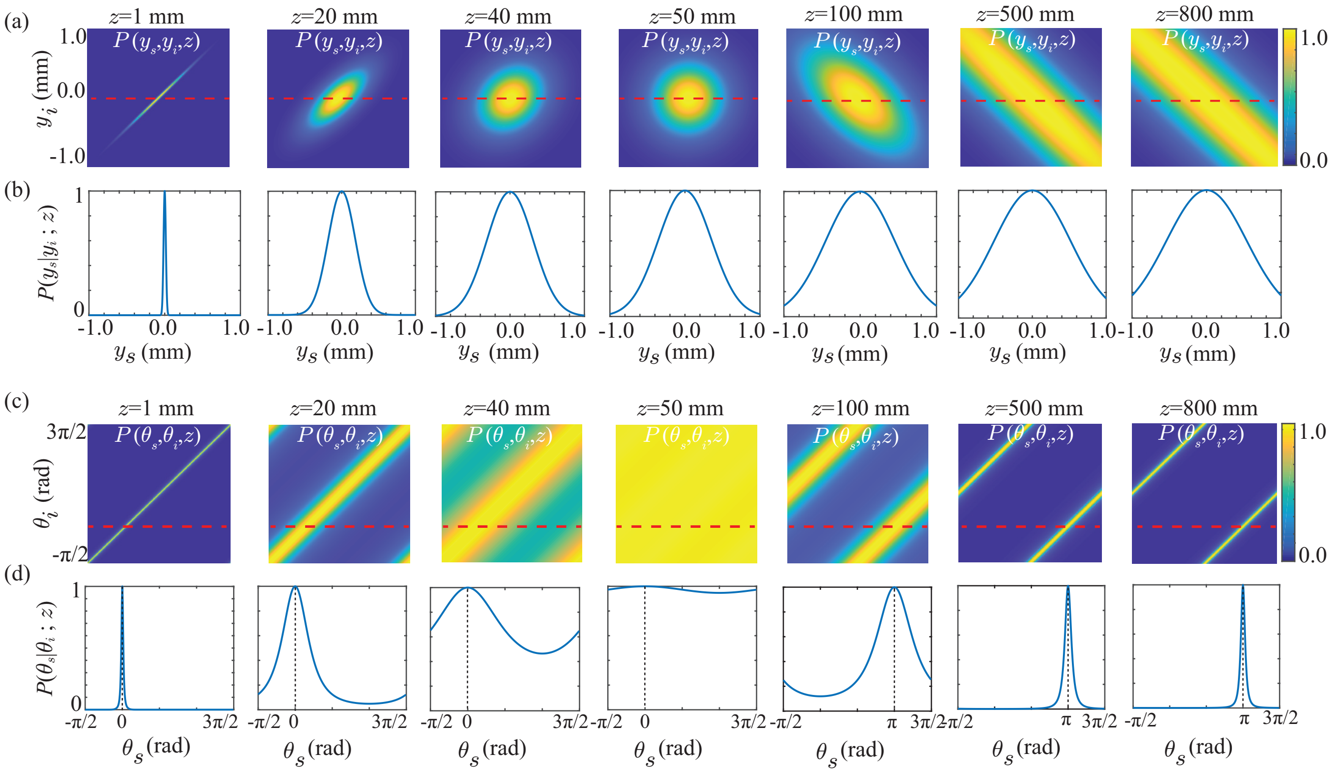

Figure S 1: **Two-photon position and angle probability distribution functions:** (a) and (c) show the two-photon position probability distribution function $P(y_s, y_i; z)$ and the angle probability distribution function $P(\theta_s, \theta_i; z)$ respectively at various z values. (b) and (d) show the conditional position probability distribution function $P(y_s|y_i; z)$ and the conditional angle probability distribution function $P(\theta_s|\theta_i; z)$ of the signal photon at various z values.

We then integrate $P(r_s, \theta_s, r_i, \theta_i; z)$ over the radial coordinates in order to obtain the two-photon angle probability distribution function $P(\theta_s, \theta_i; z)$ as:

$$P(\theta_s, \theta_i; z) = \iint r_s r_i P(r_s, \theta_s, r_i, \theta_i; z) dr_s dr_i. \tag{4}$$

As calculated using Eqs. (2) and (4), the conditional position and angle uncertainties behave differently upon propagation. Although it is very difficult to derive the general analytical expressions for the conditional position and angle uncertainties, we have obtained analytical expressions for how the conditional position and angle uncertainties scale with z in the near-

and far-field regions.

1.2 Near- and far-field behaviours of the conditional position uncertainty

The two-photon position probability distribution function is given by Eq. (2). By setting $\boldsymbol{\rho}_i = 0$, we write the conditional positional probability distribution function $P(\boldsymbol{\rho}_s|\boldsymbol{\rho}_i; z)$ as

$$P(\boldsymbol{\rho}_s|\boldsymbol{\rho}_i; z) = |A|^2 \exp\left[-\frac{\rho_s^2}{2}\left(\frac{1}{w(z)^2} + \frac{1}{\sigma(z)^2}\right)\right],$$
$$\text{where} \qquad w(z)^2 = w_0^2\left[1 + \frac{z^2}{k^2 w_0^4}\right],$$
$$\sigma(z)^2 = \sigma_0^2\left[1 + \frac{z^2}{k^2 \sigma_0^4}\right], \tag{5}$$

and $|\boldsymbol{\rho}_s|^2 = \rho_s^2$. From Eq. (5), we obtain the conditional position uncertainty in the y-direction as

$$\Delta(y_s|y_i; z) = \sqrt{\frac{1}{\frac{1}{w(z)^2} + \frac{1}{\sigma(z)^2}}}. \tag{6}$$

For the experimental parameters of interest, we have $w_0 = 507\ \mu$m and $\sigma_0 = 11.3\ \mu$m. Therefore, in the near-field region, we have $w(z) \gg \sigma(z)$ and thus the conditional uncertainty in the y-direction becomes

$$\Delta(y_s|y_i; z) \approx \sigma(z) = \sigma_0\sqrt{1 + \frac{z^2}{k^2\sigma_0^4}}. \tag{7}$$

In the far-field, we have $w(z) \ll \sigma(z)$ and thus the conditional uncertainty in the y-direction becomes

$$\Delta(y_s|y_i; z) \approx w(z) = w_0\sqrt{1 + \frac{z^2}{k^2 w_0^4}}. \tag{8}$$

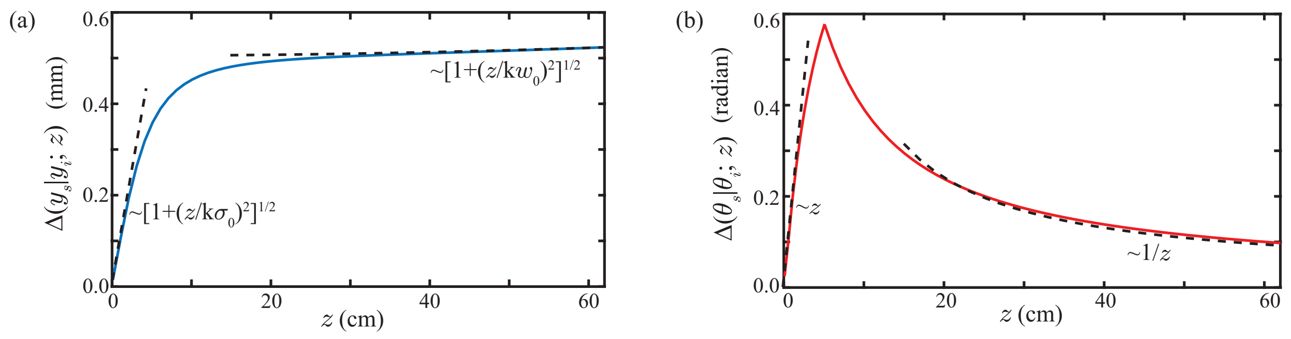

Figure S 2: **Propagation of conditional position and angle uncertainties:** (a) Numerically calculated conditional position uncertainty $\Delta(y_s|y_i;z)$ as a function of z. The two dotted lines show the z-scaling of the uncertainty in the near- and far-field regions. (b) Numerically calculated conditional angle uncertainty $\Delta(\theta_s|\theta_i;z)$ as a function of z. The two dotted lines show the z-scaling of the uncertainty in the near- and far-field regions.

1.3 Near- and far-field behaviours of the conditional angle uncertainty

The two-photon angle probability distribution function is given by Eq. (4). The conditional angle probability distribution function $P(\theta_s|\theta_i;z)$ is obtained by setting $\theta_i = 0$ in Eq. (4). As we are interested only in obtaining the near- and far-field scalings of the conditional angle uncertainty, we take $P(r_s,\theta_s,r_i,\theta_i;z) = P(r_s,\theta_s,\theta_i;z)\delta(r_s - r_i)$. Thus we write Eq. (4) as

$$P(\theta_s,\theta_i;z) = \int P(r,\theta_s,\theta_i;z)r^2 dr. \tag{9}$$

Using Eq. (3) and the Mathematica software, we evaluate the above integral and obtain

$$P(\theta_s,\theta_i;z) = \frac{P_0}{[C + D\cos(\theta_s - \theta_i)]^{3/2}}, \tag{10}$$

where

$$\begin{aligned} P_0 &= \frac{|A|^2\sqrt{\pi/2}}{8}, \\ C &= \frac{1}{2}\left[\frac{1}{w(z)^2} + \frac{1}{\sigma(z)^2}\right], \\ D &= \frac{1}{2}\left[\frac{1}{w(z)^2} - \frac{1}{\sigma(z)^2}\right]. \end{aligned} \tag{11}$$

The ratio of C and D can be written as

$$\frac{C}{D} = \frac{w(z)^2 + \sigma(z)^2}{-w(z)^2 + \sigma(z)^2} = \frac{(w_0^2 + \sigma_0^2) + \frac{z^2}{k^2}\left[\frac{1}{w_0^2} + \frac{1}{\sigma_0^2}\right]}{(-w_0^2 + \sigma_0^2) + \frac{z^2}{k^2}\left[-\frac{1}{w_0^2} + \frac{1}{\sigma_0^2}\right]}. \tag{12}$$

In our experiments, we have $w_0 = 507\ \mu$m and $\sigma_0 = 11\ \mu$m. Thus we have $w_0 \gg \sigma_0$, and under this approximation we write the above ratio as

$$\frac{C}{D} = \frac{w_0^2 + z^2/(k^2\sigma_0^2)}{-w_0^2 + z^2/(k^2\sigma_0^2)} = \frac{z^2 + k^2\sigma_0^2 w_0^2}{z^2 - k^2\sigma_0^2 w_0^2}. \tag{13}$$

Next, we study the behaviour of $P(\theta_s, \theta_i; z)$ in the near field regions. We make use of the fact that for $\theta_i = 0$, $P(\theta_s, \theta_i; z)$ is maximum at $\theta_s = 0$. Therefore, we have

$$P^{\max}(\theta_s, \theta_i = 0; z) = \frac{P_0}{[C + D]^{3/2}}. \tag{14}$$

We next find the value of θ_s at which $P(\theta_s, \theta_i; z) = P^{\max}(\theta_s, \theta_i = 0; z)/2$, in which case θ_s can be taken as the half-width of the conditional angle probability distribution function. We thus equate

$$\begin{aligned} & P(\theta_s, \theta_i; z) = P^{\max}(\theta_s, \theta_i = 0; z)/2 \\ \text{or,} \quad & \frac{P_0}{[C + D\cos\theta_s]^{3/2}} = \frac{P_0}{2[C + D]^{3/2}} \\ \text{or,} \quad & C + D\cos\theta_s = 2^{2/3}(C + D). \end{aligned} \tag{15}$$

Solving the above equation, we get two solutions for θ_s:

$$\begin{aligned} & \theta_s^{(+)} = \cos^{-1}\left[(2^{2/3} - 1)\frac{C}{D} + 2^{2/3}\right] \\ \text{and} \qquad & \theta_s^{(-)} = -\cos^{-1}\left[(2^{2/3} - 1)\frac{C}{D} + 2^{2/3}\right]. \end{aligned} \tag{16}$$

The angle uncertainty $\Delta(\theta_s|\theta_i;z)$ can therefore be written as

$$\begin{aligned}\Delta(\theta_s|\theta_i;z) &= \theta_s^{(+)} - \theta_s^{(-)}\\ &= 2\cos^{-1}\left[(2^{2/3}-1)\frac{C}{D} + 2^{2/3}\right].\end{aligned} \tag{17}$$

Using the approximation $\cos^{-1}x = \sqrt{2(1-x)}$ for $x \in [0,1]$, we write the above uncertainty as:

$$\Delta(\theta_s|\theta_i;z) = 2\sqrt{2(2^{2/3}-1)\left[-\frac{C}{D}-1\right]}. \tag{18}$$

Substituting for C/D from Eq. (12), we obtain

$$\Delta(\theta_s|\theta_i;z) = 4\sqrt{2^{2/3}-1} \times \sqrt{\frac{z^2}{k^2\sigma_0^2 w_0^2 - z^2}}. \tag{19}$$

In the near-field regions, we have $k^2\sigma_0^2 w_0^2 \gg z^2$. Therefore, we can write the angle uncertainty in the near-field regions as

$$\Delta(\theta_s|\theta_i;z) \approx \frac{4\sqrt{2^{2/3}-1}}{k\sigma_0 w_0} z. \tag{20}$$

Thus in the near-field regions the angle uncertainty increases linearly with z. In the far-field, we use the fact that for $\theta_i = 0$, $P(\theta_s, \theta_i; z)$ is maximum at $\theta_s = \pi$. Therefore, in the far-filed we have $P^{\max}(\theta_s, \theta_i = 0; z) = P_0/[C-D]^{3/2}$. Now, proceeding in the similar manner as above, and using the far-field approximation $z^2 \gg k^2\sigma_0^2 w_0^2$, we find the angle uncertainty in the far-field regions to be

$$\Delta(\theta_s|\theta_i;z) \approx 4\sqrt{2^{2/3}-1}\, k\sigma_0 w_0 \frac{1}{z}. \tag{21}$$

We thus find that in the far-field region the angle uncertainty $\Delta(\theta_s|\theta_i;z)$ becomes inversely proportional to z and as a consequence decreases upon propagation. Figure S 2(b) shows the numerically calculated conditional angle uncertainty $\Delta(\theta_s|\theta_i;z)$ as a function of z. The two dotted lines in Fig. S 2(b) show the z-scaling of the uncertainty in the near- and far-field regions.

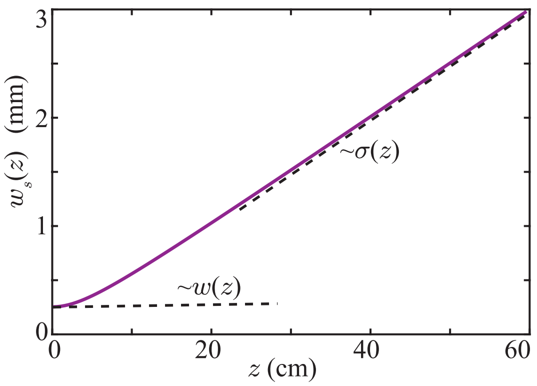

Figure S 3: Numerically calculated position uncertainty $w_s(z)$ of the signal photon as a function of z. The two dotted lines show the z-scaling of the uncertainty in the near- and far-field regions.

2 Calculation of signal photon position uncertainty

From the two-photon probability distribution in Eq. (2), we find the position probability distribution $P(\boldsymbol{\rho}_s; z)$ of the signal photon in the following manner

$$P(\boldsymbol{\rho}_s; z) = \int P(\boldsymbol{\rho}_s, \boldsymbol{\rho}_i; z) d\boldsymbol{\rho}_i = N \exp\left[-\rho_s^2/2w_s(z)^2\right], \tag{22}$$

where $w_s(z) = \Delta(y_s|y_i; z)/\sqrt{1-(D/C)^2}$ is the position uncertainty of the signal photon. We now plot $w_s(z)$ as a function of z in Fig. S 3. We find that $w_s(z)$ monotonically increases upon propagation. The two dotted lines in Fig. S 3 show the near- and far-field z scalings of $w_s(z)$. We find that in the near- and far-field $w_s(z)$ behaves as $w_s(z) \approx w(z)/2$ and $w_s(z) \approx \sigma(z)/2$, respectively.

3 Calculation of conditional momentum and OAM uncertainties

Using the two-photon wave-function in the position basis $\psi(\boldsymbol{\rho}_s, \boldsymbol{\rho}_i; z)$ calculated in the previous section, we calculate the two-photon wave-function $\psi(\boldsymbol{p}_s, \boldsymbol{p}_i; z)$ in the transverse momentum

basis, which is given by (*44*)

$$\psi(\boldsymbol{p}_s, \boldsymbol{p}_i; z) = A \exp\left[-\frac{|\boldsymbol{p}_s + \boldsymbol{p}_i|^2 w_0^2}{4\hbar^2}\right] \exp\left[-\frac{|\boldsymbol{p}_s - \boldsymbol{p}_i|^2 \sigma_0^2}{4\hbar^2}\right] \exp\left[-\frac{iz}{k\hbar^2} p_s^2 + p_i^2\right], \quad (23)$$

where $\boldsymbol{p}_s \equiv (p_{sx}, p_{sy})$ and $\boldsymbol{p}_i \equiv (p_{ix}, p_{iy})$ are the transverse momenta of the signal and idler photons, respectively. Using the above equation, we find that the conditional momentum probability distribution function $P(\boldsymbol{p}_s|\boldsymbol{p}_i; z)$ of the signal photon is given by

$$P(\boldsymbol{p}_s|\boldsymbol{p}_i; z) = A \exp\left[-\frac{p_s^2(w_0^2 + \sigma_0^2)}{2\hbar^2}\right], \quad (24)$$

where $p_s^2 = |\boldsymbol{p}_s|^2$. The standard deviation of $P(\boldsymbol{p}_s|\boldsymbol{p}_i; z)$ in the y-direction $\Delta(p_{sy}|p_{iy}; z)$ is the conditional momentum uncertainty of the signal photon. The above equation shows that $P(\boldsymbol{p}_s|\boldsymbol{p}_i; z)$ is independent of z and that $\Delta(p_{sy}|p_{iy}; z)$ does not change upon propagation.

For a Gaussian pump the two-photon state produced by SPDC in the OAM basis can be written as (*17*)

$$|\Psi\rangle = \sum_{l_s=-\infty}^{\infty} \sqrt{S_{l_s}} |l_s\rangle_s |-l_s\rangle_i, \quad (25)$$

where $l_s\hbar$ and $-l_s\hbar$ are the OAMs of signal and idler photons, respectively. The form of the two-photon state above implies that if the signal photon is detected with OAM $l_s\hbar$, then the idler photon is guaranteed to be detected with OAM $-l_s\hbar$. For the above state, and with $l_i = 0$, the conditional two-photon OAM probability distribution function takes the following form: $P(l_s|l_i; z) = S_{l_s}\delta_{l_s,0}$. This implies that the corresponding conditional OAM uncertainty $\Delta(l_s|l_i; z)$ is equal to zero. However, in an experimental situation, one always measures $\Delta(l_s|l_i; z)$ to be non-zero (*17*). There are several reasons for this, which includes the pump not being an ideal Gaussian beam, the experimental imperfections such as misalignment and background noise, and the mode dependent detection efficiencies of OAM d etectors. These cause an additional contribution in $P(l_s|l_i; z)$ measurement. Therefore, in our experiments, we model

the conditional OAM probability distribution function as:

$$P(l_s|l_i;z) = S_{l_s}\delta_{l_s,0} + N\exp\left[-\frac{l_s^2}{2\sigma_f^2}\right], \tag{26}$$

where S_0, σ_f and N are the fitting parameters. We take the width of $P(l_s|l_i;z)$ as the conditional OAM uncertainty $\Delta(l_s|l_i;z)$.

4 Coincidence Measurement with EMCCD camera

In this section we outline how we use an Andor iXon Ultra-897 EMCCD camera having 512×512 pixel grid with 16×16 μm^2 pixel-size for measuring coincidence counts in position and angle bases. For this, we record 10^6-10^7 images of the SPDC field with an exposure time of 1 ms - 5 ms over a few hours with average flux of 0.5 - 2.0 photons per pixel. We operate the camera at -60°C with the electron-multiplication gain of 1000, the horizontal pixel readout rate of 5-17 MHz, the vertical pixel shift speed of 0.3 μs, and the vertical clock amplitude of +4V. In SPDC, a signal and idler photon pair gets generated within a very short time interval, usually of the order of 100 fs, which is much smaller than the exposure time ($1-5$ ms) of the EMCCD camera. Therefore, in all likelihood, the signal and idler photons belonging to a pair arrive within the same image. However, within the same image, we can also have signal and idler photons that are not from the same down-conversion pair. These give rise to the accidental coincidences, which between the pixels (or pixel groups) p and q can be estimated by computing the coincidence counts between k^{th} and $(k+1)^{th}$ images. Therefore, as detailed in Ref. (*52,53*), the true coincidence count C_{pq} due to down-converted pairs between two pixels or two groups of pixels, p and q, of the EMCCD camera can be expressed as

$$C_{pq} = \frac{1}{N}\sum_{k=1}^{N} n_p^{(k)} n_q^{(k)} - \frac{1}{N}\sum_{k=1}^{N} n_p^{(k)} n_q^{(k+1)}, \tag{27}$$

where the first term is the total coincidence and the second term is the accidental coincidence.

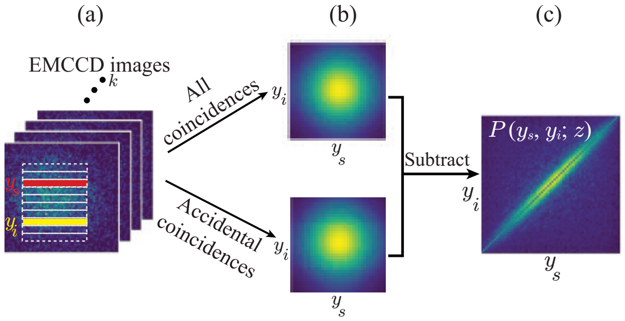

Figure S 4: **Measuring position coincidence using EMMCD:**(a) Acquired images of SPDC field and binning the pixels into signal y_s and idler y_i bars. (b) The top and the bottom images represent the total coincidence and the accidental coincidence calculated using the first and the second terms of Eq. (28), respectively. Subtraction of these two terms gives (c) the measured two-photon position probability distribution function $P(y_s, y_i; z)$.

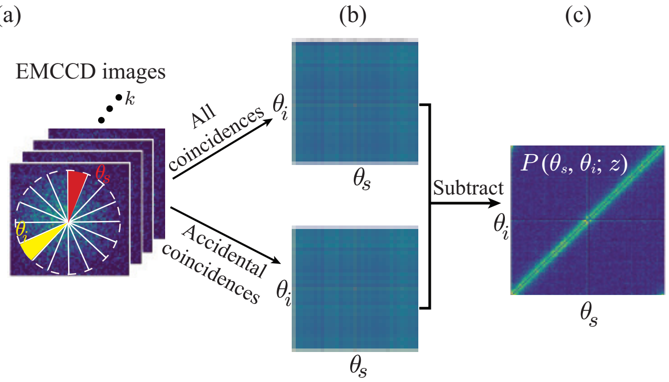

Figure S 5: **Measuring angle coincidence using EMMCD:**(a) Acquired images of SPDC field and binning the pixels into signal angular sector θ_s and idler angular sector θ_i. (b) The top and the bottom images represent the total coincidence and the accidental coincidence calculated using the first and the second terms of Eq. (29), r espectively. Subtraction of these two terms gives (c) the measured two-photon angle probability distribution function $P(\theta_s, \theta_i; z)$.

For measuring the two-photon position probability distribution function $P(y_s, y_i; z)$, we take millions of images using the EMCCD camera. For each image, we group the pixels into horizontal strips, y_s and y_i, as shown in Fig. S 4(a). The coincidence count between y_s and y_i can be written using Eq. (27) as

$$C_{y_s y_i} = \frac{1}{N}\sum_{k=1}^{N} n_{y_s}^{(k)} n_{y_i}^{(k)} - \frac{1}{N}\sum_{k=1}^{N} n_{y_s}^{(k)} n_{y_i}^{(k+1)}, \tag{28}$$

where $n_{y_s}^{(k)}$ and $n_{y_i}^{(k)}$ are the photon counts of y_s and y_i respectively. The top image in Fig. S 4(b) represents total coincidence as a function of y_s and y_i, and it is evaluated by using the first term of Eq. (28). The bottom image in Fig. S 4(b) represents the accidental coincidence as a function of y_s and y_i, and it is evaluated using the second term of Eq. (28). The difference of these two images is proportional to the true coincidence $C_{y_s y_i}$ and thus to the two-photon position probability distribution function $P(y_s, y_i; z)$, as shown in Fig. S 4(c). At $y_s = y_i$, the correlation becomes artificially perfect because we are correlating a pixel with itself. So, we discard the points with $y_s = y_i$ as outliers.

For measuring the two-photon angle probability distribution $P(\theta_s, \theta_i; z)$, we group the pixels for each image into angular sectors as shown in Fig. S 5(a). The coincidence count between the angular sectors at θ_s and θ_i is given by:

$$C_{\theta_s \theta_i} = \frac{1}{N}\sum_{k=1}^{N} n_{\theta_s}^{(k)} n_{\theta_i}^{(k)} - \frac{1}{N}\sum_{k=1}^{N} n_{\theta_s}^{(k)} n_{\theta_i}^{(k+1)}, \tag{29}$$

where $n_{\theta_s}^{(k)}$ and $n_{\theta_i}^{(k)}$ are the photon counts of angular sectors θ_s and θ_i respectively. The top image in Fig. S 5(b) represents the total coincidence as a function of θ_s and θ_i, and it is evaluated using the first term of Eq. (29). The bottom image in Fig. S 5(b) represents the accidental coincidence as a function of θ_s and θ_i, and it is evaluated using the second term of Eq. (29). The difference of these two images is proportional to the two-photon angle probability distribution $P(\theta_s, \theta_i; z)$, as shown in Fig. S 5(c). At $\theta_s = \theta_i$, the correlation becomes artificially perfect because we are correlating a pixel with itself. So, we discard the points with $\theta_s = \theta_i$ as outliers.

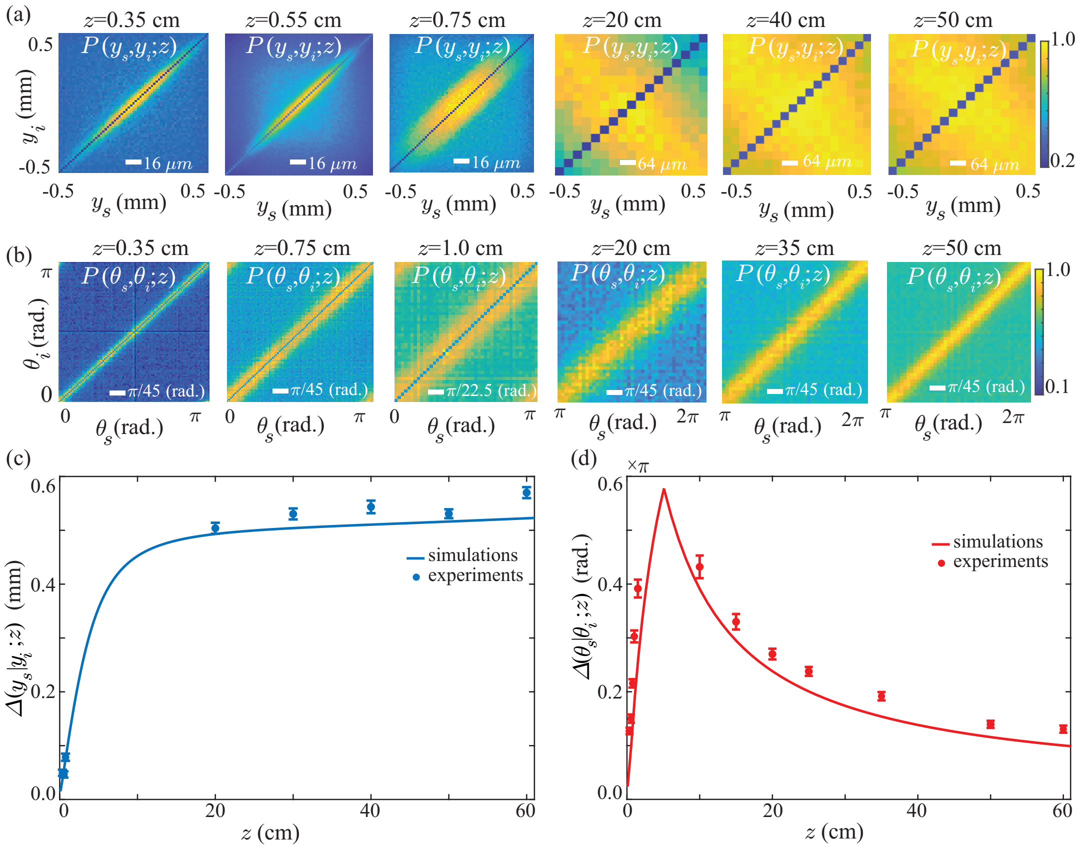

Figure S 6: **Experimentally measured two-photon position and angle correlations:** (a) and (b) show the experimentally measured two-photon position and angle probability distributions $P(y_s, y_i; z)$ and $P(\theta_s, \theta_i; z)$ as a function of the propagation distance z. (c) and (d) show the plots of the position and angle uncertainties $\Delta(y_s|y_i; z)$ and $\Delta(\theta_s|\theta_i; z)$ as a function of z. The experimental points are shown with solid dots while the solid curves represent the numerical simulations.

5 Measurement of the two-photon position and angle probability distribution functions

Figure S 6(a) shows the experimentally measured two-photon position probability distribution function $P(y_s, y_i; z)$ at different z. For imaging the transverse planes between $z = 0.35$ cm and $z = 1.5$ cm, we keep the magnification of the imaging system to be 1 while for imaging the transverse planes between $z = 10$ and $z = 60$ cm, we keep the magnification to

be 0.25. We scale the measured $P(y_s, y_i; z)$ such that its maximum value is equal to one. We find that the photons are correlated in position in the near field whereas they get position anti-correlated in the far-field. In order to extract $\Delta(y_s|y_i; z)$ from the measured $P(y_s, y_i; z)$, we fit $P(y_s, y_i; z)$ with the function: $P_f(y_s, y_i; z) = bP_r(y_s, y_i; z) + aP_n(y_s, y_i; z)$, where $P_r(y_s, y_i; z) = \exp[-(y_s + y_i - d)^2/(2\sigma_1^2(z))] \times \exp[-(y_s - y_i - f)^2/(2\sigma_2^2(z))]$ is considered as the probability distribution due to the down-converted photons, while $P_n(y_s, y_i; z) = \exp[-(y_s + y_i - d)^2/(2n^2)] \times \exp[-(y_s - y_i - f)^2/(2m^2)]$ is considered as the noise contribution. Here b, a, $\sigma_1(z)$, $\sigma_2(z)$, d, f, m and n are the fitting parameters. We consider $n \gg \sigma_1(z)$, $m \gg \sigma_2(z)$ such that the noise contribution remains much broader than the two-photon position probability distribution. The width $\Delta(y_s|y_i = 0; z)$ can now be expressed as $\Delta(y_s|y_i = 0; z) = \sigma_1(z)\sigma_2(z)/\sqrt{\sigma_1^2(z) + \sigma_2^2(z)}$. Figure S 6(c) shows $\Delta(y_s|y_i = 0; z)$ as a function of z. The simulation has been carried out using the expression given in Eq. (2).

Figure S 6(b) shows the experimentally measured $P(\theta_s, \theta_i; z)$ at different z. We scale $P(\theta_s, \theta_i; z)$ such that its maximum value is equal to one. The $P(\theta_s, \theta_i; z)$ plots show that near the crystal plane the signal and idler photons have the highest probability of arriving at the same angular positions. However in the far-field the signal and idler photons have the highest probability of arriving at angular positions separated by π radians. We fit the measured $P(\theta_s, \theta_i; z)$ with the analytic function: $P_f(\theta_s, \theta_i; z) = bP_r(\theta_s, \theta_i; z) + a$, where $P_r(\theta_s, \theta_i; z) = 1/(1 + q\cos(\theta_s - \theta_i - c))^{3/2}$. Here, b, a, q, and c, are the fitting parameters. We derive the fitting function by putting $r_s = r_i$ in Eq. (4). Next, we evaluate $\Delta(\theta_s|\theta_i; z)$ by finding the standard deviation of $P_r(\theta_s|\theta_i; z)$ at various z values. Figure S 6(d) shows the experimental $\Delta(\theta_s|\theta_i; z)$ as a function of z. We find that near the crystal $\Delta(\theta_s|\theta_i; z)$ increases as a function of z. However, beyond $z = 10$ cm, $\Delta(\theta_s|\theta_i; z)$ starts to monotonically decreas as a function of z. The numerical simulations have been carried out using the expression given in Eq. (4).

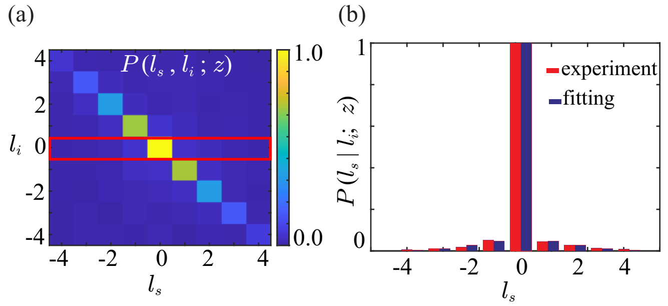

Figure S 7: **Experimentally measured two-photon OAM correlation:** (a) and (b) are the experimentally measured $P(l_s, l_i; z)$ and $P(l_s|l_i; z)$ at $z = 50$ cm. The fitting is based on the noise model described in Eq. (26).

6 Measurement of the two-photon momentum and OAM probability distribution functions

Equation (24) shows that $P(\boldsymbol{p}_s|\boldsymbol{p}_i; z)$ is independent of z and that $\Delta(p_{sy}|p_{iy}; z)$ does not change upon propagation. For the given experimental parameters, the calculated value of $\Delta(p_{sy}|p_{iy}; z)$ is $1.97\hbar$ mm $^{-1}$. The two-photon OAM probability distribution function $P(l_s, l_i; z)$ remains unchanged as a function of z. We verify this by making several measurements of $P(l_s, l_i; z)$ as a function of z. We plot the experimentally measured two-photon OAM probability distribution function $P(l_s, l_i; z)$ and the conditional OAM probability distribution function $P(l_s|l_i; z)$ at $z = 50$ cm in Figs. S 7(a) and S 7(b), respectively. As described in Sec. 3, we fit the conditional distribution with the analytical function $P(l_s|l_i; z) = S_{l_s}\delta_{l_s,0} + N \exp\left[-l_s^2/(2\sigma_f^2)\right]$, where S_0, N and σ_f are the fitting parameters, and thus find the uncertainty $\Delta(l_s|l_i; z)$ to be $0.72\hbar$ radian^{-1} in our experiments.

7 Measurement of the two-photon angle probability distribution function in turbulence

In this section, we present how the conditional angle uncertainty propagates after the two-photon field passes through turbulence. Figure S 8(a) illustrates the propagation of SPDC photons through a planar turbulence kept at a distance $z = d$ from the crystal plane located at $z = 0$. We are interested in finding the two-photon angle probability distribution function at a propagation distance z. The presence of turbulence introduces statistical randomness in the two-photon field, and so we need to describe the field propagation in terms of the two-photon cross-spectral density function. From $z = 0$ up to $z = d$, the two photon field remains pure and can be described by the two-photon wave-function $\psi(\boldsymbol{\rho}_s, \boldsymbol{\rho}_i; z)$. Therefore, the two-photon cross-spectral density function $W(\boldsymbol{\rho}'_{s1}, \boldsymbol{\rho}'_{i1}, \boldsymbol{\rho}'_{s2}, \boldsymbol{\rho}'_{i2}; z_t)$ right after the turbulence plane $z = d$ can be written as

$$W(\boldsymbol{\rho}'_{s1}, \boldsymbol{\rho}'_{i1}, \boldsymbol{\rho}'_{s2}, \boldsymbol{\rho}'_{i2}; d) = \psi^*(\boldsymbol{\rho}'_{s2}, \boldsymbol{\rho}'_{i2}; d)\psi(\boldsymbol{\rho}'_{s1}, \boldsymbol{\rho}'_{i1}; d) \times W_{\text{turb}}(\boldsymbol{\rho}'_{s1}, \boldsymbol{\rho}'_{s2}, \boldsymbol{\rho}'_{i1}, \boldsymbol{\rho}'_{i2}), \tag{30}$$

where, $\boldsymbol{\rho}'_s \equiv (x'_s, y'_s)$, and $\boldsymbol{\rho}'_i \equiv (x'_i, y'_i)$ are the transverse co-ordinates of signal and idler photons respectively, at $z = d$. The term $\psi^*(\boldsymbol{\rho}'_{s2}, \boldsymbol{\rho}'_{i2}; d)\psi(\boldsymbol{\rho}'_{s1}, \boldsymbol{\rho}'_{i1}; d)$ is the two-photon cross spectral density function right before the turbulence plane. The effect due to the turbulence is captured through the cross-spectral density function $W_{\text{turb}}(\boldsymbol{\rho}'_{s1}, \boldsymbol{\rho}'_{s2}, \boldsymbol{\rho}'_{i1}, \boldsymbol{\rho}'_{i2})$, which we approximate by modelling the turbulence in terms of a Gaussian function

$$W_{\text{turb}}(\boldsymbol{\rho}'_{s1}, \boldsymbol{\rho}'_{s2}, \boldsymbol{\rho}'_{i1}, \boldsymbol{\rho}'_{i2}) = \exp\left[-[|\boldsymbol{\rho}'_{s2} - \boldsymbol{\rho}'_{s1}|^2 + |\boldsymbol{\rho}'_{i2} - \boldsymbol{\rho}'_{i1}|^2]/(2r^2)\right], \tag{31}$$

where r is the turbulence strength (*55*). We rewrite the above equation as

$$W(\boldsymbol{\rho}'_{s1}, \boldsymbol{\rho}'_{i1}, \boldsymbol{\rho}'_{s2}, \boldsymbol{\rho}'_{i2}; d) = W_{\text{turb}}(\boldsymbol{\rho}'_{s1}, \boldsymbol{\rho}'_{s2}, \boldsymbol{\rho}'_{i1}, \boldsymbol{\rho}'_{i2}) e^{\frac{ik}{2d}(\rho'^2_{s2}+\rho'^2_{i2}-\rho'^2_{s1}-\rho'^2_{i1})} \int \psi^*(\boldsymbol{\rho}''_{s1}, \boldsymbol{\rho}''_{i1}; 0)$$
$$\times \psi(\boldsymbol{\rho}''_{s2}, \boldsymbol{\rho}''_{i2}; 0) e^{\frac{ik}{2d}(\rho''^2_{s2}+\rho''^2_{i2}-\rho''^2_{s1}-\rho''^2_{i1})} e^{-\frac{ik}{d}(\boldsymbol{\rho}'_{s2}\cdot\boldsymbol{\rho}''_{s2}-\boldsymbol{\rho}'_{s1}\cdot\boldsymbol{\rho}''_{s1})}$$
$$\times e^{-\frac{ik}{d}(\boldsymbol{\rho}'_{i2}\cdot\boldsymbol{\rho}''_{i2}-\boldsymbol{\rho}'_{i1}\cdot\boldsymbol{\rho}''_{i1})} d\boldsymbol{\rho}''_{s2} d\boldsymbol{\rho}''_{s1} d\boldsymbol{\rho}''_{i2} d\boldsymbol{\rho}''_{i1}, \tag{32}$$

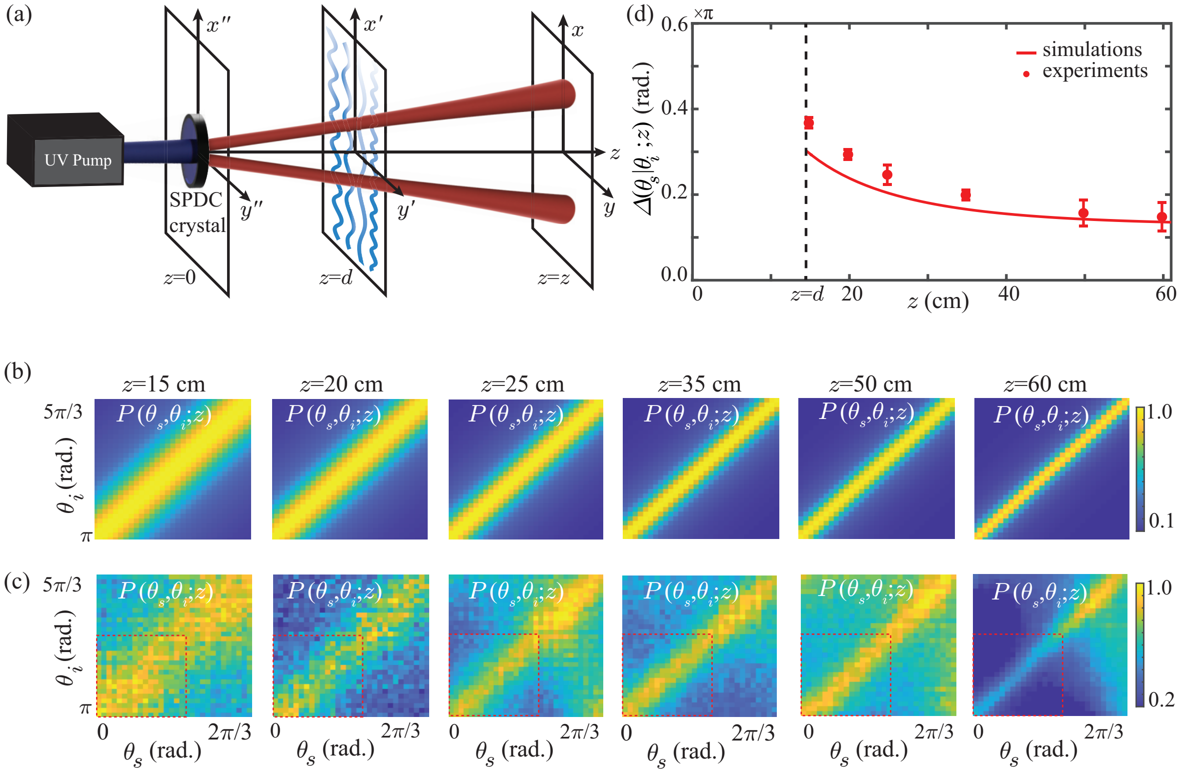

Figure S 8: **Two-photon angle correlation in turbulence:**(a) Illustrating the propagation of the down-converted field in the presence of turbulence. (b) Numerically calculated and (c) experimentally measured two-photon angle probability distribution function $P(\theta_s, \theta_i; z)$ at various z in the presence of turbulence. (d) The numerical and experimental plots of $\Delta(\theta_s|\theta_i; z)$ as a function of z.

where $\psi(\boldsymbol{\rho}''_s, \boldsymbol{\rho}''_i; 0)$ is the two-photon wave-function at the crystal plane $z = 0$ and is given by Eq. (1). Now, by propagating $W(\boldsymbol{\rho}'_{s1}, \boldsymbol{\rho}'_{i1}, \boldsymbol{\rho}'_{s2}, \boldsymbol{\rho}'_{i2}; d)$ from $z = d$ up to $z = z$, we find the two-photon cross-spectral density function at z and thereby the two-photon position probability distribution function $P(\boldsymbol{\rho}_s, \boldsymbol{\rho}_i; z)$:

$$P(\boldsymbol{\rho}_s, \boldsymbol{\rho}_i; z) = \int W(\boldsymbol{\rho}'_{s1}, \boldsymbol{\rho}'_{i1}, \boldsymbol{\rho}'_{s2}, \boldsymbol{\rho}'_{i2}; z_t) e^{\frac{ik}{2(z-d)}(\rho'^2_{s2}+\rho'^2_{i2})} e^{-\frac{ik}{2(z-d)}(\rho'^2_{s1}+\rho'^2_{i1})} e^{-\frac{ik}{(z-d)}\boldsymbol{\rho}_s\cdot(\boldsymbol{\rho}'_{s2}-\boldsymbol{\rho}'_{s1})}$$
$$\times\, e^{\frac{ik}{(z-d)}\boldsymbol{\rho}_i\cdot(\boldsymbol{\rho}'_{i2}-\boldsymbol{\rho}'_{i1})} d\boldsymbol{\rho}'_{s2} d\boldsymbol{\rho}'_{s1} d\boldsymbol{\rho}'_{i2} d\boldsymbol{\rho}'_{i1}. \quad (33)$$

By substituting Eq. (32) into Eq. (33), we compute $P(\boldsymbol{\rho}_s, \boldsymbol{\rho}_i; z)$ as a function of z. We then use the transformations $\boldsymbol{\rho}_s = (r_s \cos\theta_s,\, r_s \sin\theta_s)$ and $\boldsymbol{\rho}_i = (r_i \cos\theta_i,\, r_i \sin\theta_i)$ in order to obtain

$P(\theta_s, \theta_i; z)$ using Eq. (33).

Figure S 8(c) shows the experimentally measured $P(\theta_s, \theta_i; z)$ at different z (see Sec. 5 for the measurement details). Figure S 8(b) shows the $P(\theta_s, \theta_i; z)$ calculated using Eq. (4) at different z for the relevant experimental parameters of $d = 15$ cm, $L = 5$ mm, $w_0 = 507$ μm. For the simulation plots, we use the turbulence strength r as a fitting parameter and find its value to be 0.125 mm. We note that the experimentally measured $P(\theta_s, \theta_i; z)$ contains some noise distribution, which gets prominent at large z. This can be attributed to the fact that in the experiment, we insert a distributed turbulence in the path of the two-photon field, whereas in the simulations, we approximate that as a planar turbulence. Nevertheless, the diagonal correlation in the experimentally observed $P(\theta_s, \theta_i; z)$ matches with that of the numerical simulations. Using the procedure described in Sec. 5 B, we extract the conditional angle uncertainty $\Delta(\theta_s|\theta_i; z)$ from Figure S 8(c) and plot them in Figure S 8(d). In order to minimize the effect of noise distribution on the estimation of $\Delta(\theta_s|\theta_i; z)$, we select a region of $P(\theta_s, \theta_i; z)$ as shown by the dotted red box in Fig. S 8(c).

8 Measurement of the two-photon OAM probability distribution in turbulence

In this section, we present a theoretical model to evaluate the influence of turbulence on the conditional OAM distribution $P(l_s|l_i; z)$. Within paraxial approximation and the Gaussian pump beam assumption (*17*), the OAM remains conserved in SPDC. This means that if the idler photon is detected with OAM $l_i\hbar = 0$, the signal photon is guaranteed to be detected with OAM $l_s\hbar = 0$. For the measurement of conditional OAM distribution we choose $l_i = 0$. Therefore the signal mode can be represented as: $\psi_s(\boldsymbol{\rho}'_s; d) = \exp\left[-\rho'^2_s/4\sigma_r^2\right]$. For evaluating the influence of turbulence on the conditional OAM distribution $P(l_s|l_i = 0; z)$ of the signal photon, we simply need to evaluate how the Gaussian mode $\psi_s(\boldsymbol{\rho}'_s; d) = \exp\left[-\rho'^2_s/4\sigma_r^2\right]$ gets

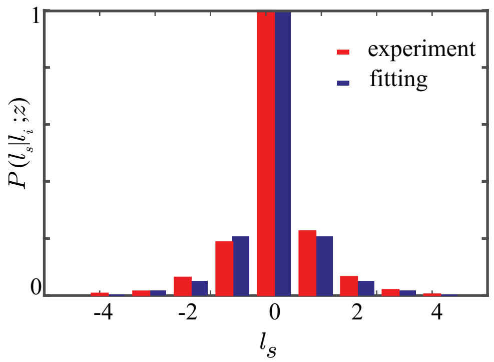

Figure S 9: The experimentally measured $P(l_s|l_i; z)$ at $z = 50$ cm in turbulence. The fitting is based on the noise model described in Eq. (37).

affected by turbulence. For this purpose, we calculate the cross-spectral density function of the signal photon right after the turbulence plane $z = d$ [see Fig. S 8(a)]. From $z = 0$ up to $z = d$, the signal field $\psi_s(\boldsymbol{\rho}'_s; d)$ remains pure. Therefore, the cross-spectral density function $W(\boldsymbol{\rho}'_{s2}, \boldsymbol{\rho}'_{s1}; d)$ right after the turbulence plane $z = d$ can be written as $W(\boldsymbol{\rho}'_{s2}, \boldsymbol{\rho}'_{s1}; d) = \psi^*(\boldsymbol{\rho}'_{s2}; d)\psi(\boldsymbol{\rho}'_{s1}; d)W_{\text{turb}}(\boldsymbol{\rho}'_{s2}, \boldsymbol{\rho}'_{s1})$, where $\boldsymbol{\rho}'_s \equiv (x'_s, y'_s)$, is the transverse co-ordinates of signal photon at $z = d$ plane. The term $\psi^*(\boldsymbol{\rho}'_{s2}; d)\psi(\boldsymbol{\rho}'_{s1}; d)$ is the cross spectral density function of the signal photon at $z = d$ right before the turbulence plane. $W_{\text{turb}}(\boldsymbol{\rho}'_{s2}, \boldsymbol{\rho}'_{s1})$ is the cross-spectral density introduced by the turbulence. We approximate it as $W_{\text{turb}}(\boldsymbol{\rho}'_{s2}, \boldsymbol{\rho}'_{s1}) = \exp\left[-|\boldsymbol{\rho}'_{s2} - \boldsymbol{\rho}'_{s1}|^2/(2r^2)\right]$, where r is the turbulence strength. Therefore, we have

$$W(\boldsymbol{\rho}'_{s2}, \boldsymbol{\rho}'_{s1}; d) = \exp\left[-\frac{\rho'^2_{s1} + \rho'^2_{s2}}{4\sigma_r^2}\right] \exp\left[-\frac{|\boldsymbol{\rho}'_{s2} - \boldsymbol{\rho}'_{s1}|^2}{2r^2}\right]. \tag{34}$$

Now, by propagating the above cross-spectral density function from $z = d$ to $z = z$, we obtain the cross-spectral density function $W(\boldsymbol{\rho}_{s1}, \boldsymbol{\rho}_{s2}; z)$ at $z = z$

$$W(\boldsymbol{\rho}_{s1}, \boldsymbol{\rho}_{s2}; z) = \exp\left[-\frac{ik}{2R(z)}(\rho^2_{s2} - \rho^2_{s1})\right] \exp\left[-\frac{\rho^2_{s1} + \rho^2_{s2}}{4\sigma_r^2(z)}\right] \exp\left[-\frac{\Delta\rho_s^2}{2r^2(z)}\right], \tag{35}$$

where $\Delta\rho_s = |\boldsymbol{\rho}_2 - \boldsymbol{\rho}_1|$, $r(z) = r\sqrt{1 + \left(\frac{z-z_t}{k_s\sigma_r\delta}\right)^2}$, $\sigma_r(z) = \sigma_r\sqrt{1 + \left(\frac{z-z_t}{k_s\sigma_r\delta}\right)^2}$, $\frac{1}{\delta^2} = \frac{1}{r^2} + \frac{1}{4\sigma_r^2}$, and $k_s = \pi/\lambda_p$. We use the transformation $\boldsymbol{\rho}_{s1} \equiv (r_{s1}\cos\theta_{s1}, r_{s1}\sin\theta_{s1})$ and $\boldsymbol{\rho}_{s2} \equiv$

$(r_{s2}\cos\theta_{s2}, r_{s2}\sin\theta_{s2})$ in order to write $W(\boldsymbol{\rho}_{s1}, \boldsymbol{\rho}_{s2}; z)$ as $W_s(r_s, \theta_{s1}, \theta_{s2}; z)$. The OAM distribution of the signal photon is same as the conditional distribution $P(l_s|l_i; z)$, which we write as

$$P(l_s|l_i, z) = \iiint r_s W_s(r_s, \theta_{s1}, \theta_{s2}; z) e^{il_s(\theta_{s2}-\theta_{s1})} dr_s d\theta_{s1} d\theta_{s2}. \tag{36}$$

We compute the above integral numerically and find that it very closely fits the function $a\exp\left[-b|l_s|\right]$, where a and b are fitting parameters. For reasons described in section 3, we have added the noise term $N\exp\left[-\frac{l_s^2}{2\sigma_f^2}\right]$ in Eq. (36). We thus write the conditional OAM distribution as

$$P(l_s|l_i, z) = a\exp\left[-b|l_s|\right] + N\exp\left[-\frac{l_s^2}{2\sigma_f^2}\right]. \tag{37}$$

Figure S 9 shows the experimentally measured $P(l_s|l_i; z)$ at $z = 50$ cm. We fit $P(l_s|l_i; z = 50)$ with Eq. (37) and obtain the experimental uncertainty $\Delta(l_s|l_i; z)$ to be $0.94\hbar$ radian^{-1}.

9 Effects of a finite-size detector on angle-OAM entanglement

In our analysis so far, we have considered the photon detectors to have an aperture of infinite extent, as we take the upper limit of radial integration to be ∞ in Eq. (4). However, experiments are always carried out with detectors of finite extent. In this section, we numerically analyze the effects of using finite-size detectors on the revival of entanglement in the angle-OAM entanglement bases. The two-photon angle probability distribution function $P(\theta_s, \theta_i; z)$ in this case is given by

$$P(\theta_s, \theta_i; z) = \int_0^{D/2}\int_0^{D/2} r_s r_i P(r_s, \theta_s, r_i, \theta_i; z) dr_s dr_i, \tag{38}$$

where D represents the diameter of the detector. Using the above equation, we numerically calculate $\Delta(\theta_s|\theta_i; z)$ as a function of z upto a propagation distance of $z = 1$ km for different values of D. We use the conditional OAM uncertainty $\Delta(l_s|l_i; z) = 0.72\hbar$ corresponding to

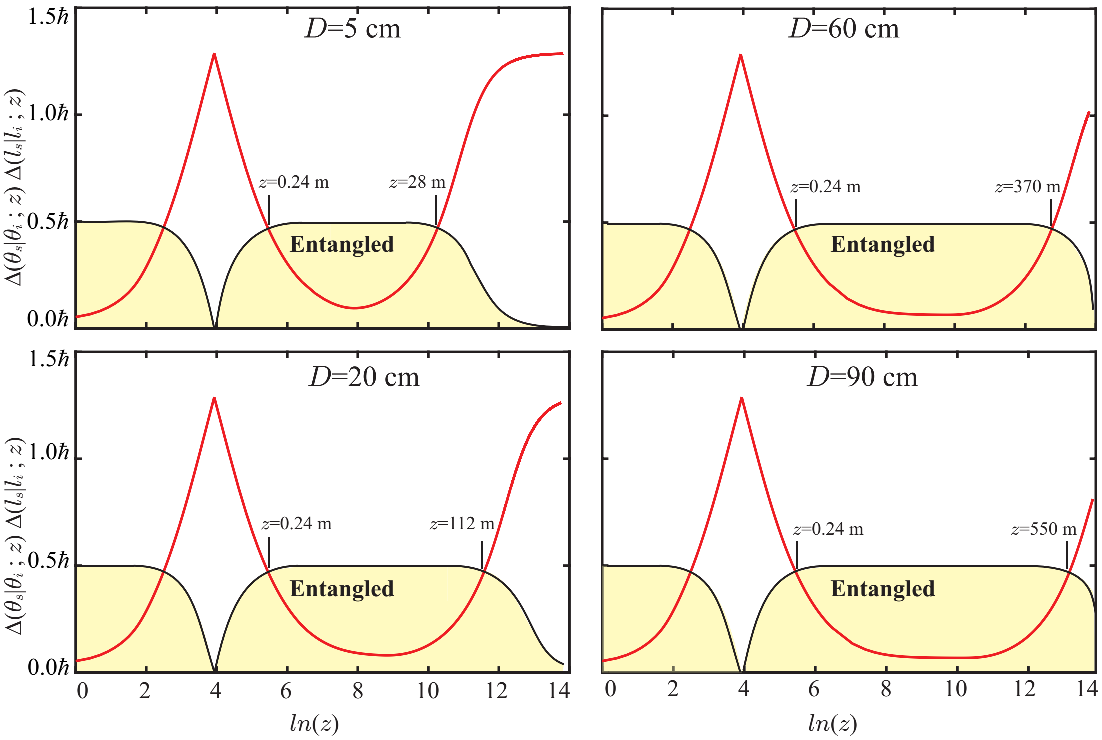

Figure S 10: Conditional angle-OAM uncertainty product $\Delta(\theta_s|\theta_i;z)\Delta(l_s|l_i;z)$ as a function of $ln(z)$ for different values of the aperture size D.

probability distribution given in Eq. (26). Here we assume that $\Delta(l_s|l_i;z)$ is not affected by the finite aperture s ize. In order to show the behaviour of $\Delta(\theta_s|\theta_i;z)\Delta(l_s|l_i;z)$ from $z = 1$ mm to 1 km, we plot $\Delta(\theta_s|\theta_i;z)\Delta(l_s|l_i;z)$ as a function of $ln(z)$ using the relevant experimental parameters. We see that for all values of D, the entanglement revives at $z = 24$ cm. We find that due to finite D, the product $\Delta(\theta_s|\theta_i;z)\Delta(l_s|l_i;z)$ starts increasing upon long-distance propagation and beyond a certain z the entanglement is not witnessed in the angle-OAM bases. Furthermore, we find that the increase in diameter D increases the propagation distance upto which the entanglement in the angle-OAM bases remains intact. Therefore, one can decide the minimum aperture size for a given application based on the distance upto which one needs the entanglement in the angle-OAM bases to be intact.

10 Witnessing revival in the angle-OAM basis through Entanglement of Formation

The entanglement of formation (EOF) has been recently introduced in Ref. (*46*) for certifying continuous-variable entanglement in position-momentum, angle-OAM, and time-energy bases. Some recent experiments have used this quantity for certifying position-momentum entanglement (*47*). In this section, we show that in addition to EPR correlation product, the revival feature in the angle-OAM bases can also be witnessed through EOF. The minimum value $E_f^{(min)}$ of EOF is related to the conditional Shannon entropies in the angle-OAM bases as (*46*)

$$E_f^{(min)}(z) = log(2\pi) - H(l_s|l_i; z) - h(\theta_s|\theta_i; z), \tag{39}$$

where

$$h(\theta_s|\theta_i; z) = h(\theta_s, \theta_i; z) - h(\theta_i; z) = -\int P(\theta_s, \theta_i; z) \log P(\theta_s, \theta_i; z) d\theta_s d\theta_i + \int P(\theta_i; z) \times \log P(\theta_i; z) d\theta_i$$

and

$$H(l_s|l_i; z) = H(l_s, l_i; z) - H(l_i; z) = -\sum_{l_s, l_i} P(l_s, l_i; z) \log P(l_s, l_i; z) + \sum_{l_i} P(l_i; z) \log P(l_i; z) \tag{40}$$

are the conditional Shannon entropies in the angle and OAM bases, respectively. $E_f^{(min)}$ lies between 0 and ∞. $E_f^{(min)} > 0$ implies the presence of entanglement while $E_f^{(min)} = 0$ implies the absence of entanglement. We use $P(\theta_i; z) = \int P(\theta_s, \theta_i; z) d\theta_s$, where $P(\theta_s, \theta_i; z)$ is given in Eq. (4), and calculate $h(\theta_s|\theta_i; z)$ using the relevent experimental parameters. We next use Eqs. (25) and (26) for writing the following OAM distributions:

$$P(l_s, l_i; z) = S_{l_s} \delta_{l_s, -l_i} + N \exp\left[-\frac{(l_s + l_i)^2}{2\sigma_f^2}\right]$$

$$\text{and} \qquad P(l_s, l_i; z) = \sum_{l_s} P(l_s, l_i; z). \tag{41}$$

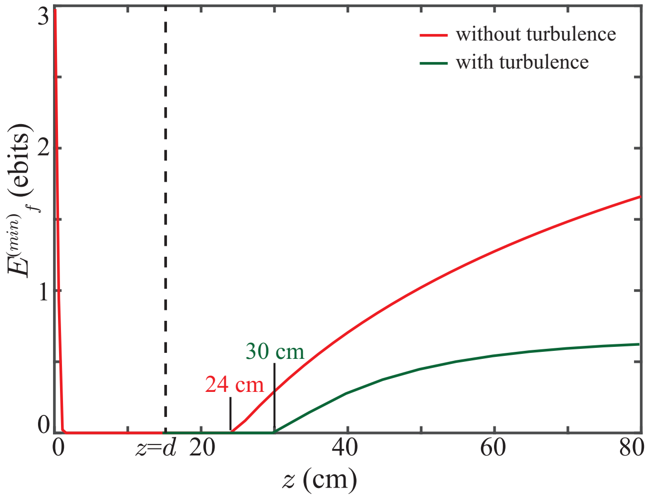

Figure S 11: Minimum value $E_f^{(min)}$ of entanglement of formation in the angle-OAM bases as a function of z with and without turbulence.

Using the above probability distributions, we calculate the conditional OAM entropy $H(l_s|l_i;z)$ and finally plot $E_f^{min}(z)$ as a function of z in Fig. S 11 (solid red curve). We find that $E_f^{(min)}$ decreases rapidly upon propagation and reaches zero within a propagation distance of 1 cm from the crystal. However, $E_f^{(min)}$ revives and becomes positive beyond $z = 24$ cm.

We next calculate EOF in the presence of turbulence. In order to calculate $h(\theta_s|\theta_i;z)$ in turbulence, we use Eq. (33) for $P(\theta_s,\theta_i;z)$ and $P(\theta_i;z) = \int P(\theta_s,\theta_i;z)d\theta_s$ for idler photon angle distribution. We then follow the model presented in Sec. 8 and use Eq. (37) for writing $P(l_s,l_i;z)$ in the turbulence which is given by

$$P(l_s,l_i;z) = S_{l_s} \exp\left[-b|l_s+l_i|\right] + N \exp\left[-\frac{(l_s+l_i)^2}{2\sigma_f^2}\right]. \tag{42}$$

We write the corresponding idler OAM distribution as $P(l_i;z) = \sum_{l_s} P(l_s,l_i;z)$, and using $P(l_s,l_i;z)$ and $P(l_i;z)$, we calculate $H(\theta_s|\theta_i;z)$. Figure S 11 (green solid line) shows $E_f^{(min)}$ as a function of z. We find that in the presence of turbulence the revival takes place at $z = 30$ cm. Thus we see that the revival of entanglement in the angle-OAM bases can also be witnessed through entanglement of formation.
